# Bromoform

**Published:** 1899-02-04

**Authors:** 


### BROMOFORM.

The growing popularity of bromoform as a means of checking the cough and vomiting of whooping-cough gives interest to certain cases of poisoning by the drug which have recently occurred. It appears that in some of the cases reported it was the last dose in the bottle of mixture that did the harm, and it is evident that the accident happened from the bromoform separating and sinking to the bottom of the bottle owing to its high specific gravity. Bromoform is nearly three times as heavy as water, and if made into a mixture has to be suspended in an emulsion. The result is that, unless the greatest care is taken to thoroughly shake the bottle each time the medicine is administered, the mixture is not complete, and an undue proportion of the drug is accumulated in the last dose. One way of getting over this difficulty is to give directions that the last dose shall always be thrown away. This plan, however, is not only wasteful but presupposes that the earlier doses have been deficient in strength. Perhaps a better plan is to arrange for each dose to be separately measured out from a drop bottle when it is required, and taken in half a teaspoonful of glycerine. Or it may be given in capsules, each containing half a minim. Bromoform is soluble in alcohol and ether, but children take it better in glycerine or in an emulsion than they do in spirit. The following is the prescription used by Marfan: Bromoform, 48 drops; almond oil, fl. dr. 5; tragacanth, fl. dr. ½; gum arabic, fl. dr. 1; cherry laurel water, fl. dr. 1; water to fl. oz. 3. Add the bromoform to the oil and make an emulsion with the gums; fl. dr. 1 contains min. 2. The dose he gives is 4 drops during the 24 hours for each year of the child's life.

